# Innate lymphoid cells and parasites: Ancient foes with shared history

**DOI:** 10.1111/pim.12513

**Published:** 2018-01-29

**Authors:** D. R. Neill, P. G. Fallon

**Affiliations:** ^1^ Institute of Infection and Global Health University of Liverpool Liverpool UK; ^2^ Trinity Biomedical Sciences Institute School of Medicine Trinity College Dublin Dublin Ireland

**Keywords:** innate immunity, Innate lymphoid cells, Host‐parasite interaction, mucosal immunity

## Abstract

This special issue of *Parasite Immunology* charts the rapid advances made in our understanding of the myriad interactions between innate lymphoid cells and parasites and how these interactions have shaped our evolutionary history. Here, we provide an overview of the issue and highlight key findings from studies in mice and man.

Innate lymphoid cells (ILCs), lacking antigen receptors but derived from the lymphoid lineage, are recently described new players in immunity that have fundamental roles in immune homeostasis, response to infection, allergy and autoimmunity. Recent years have seen global efforts to unravel the complexities of ILC evolution, lineage commitment and functionality and explore opportunities for manipulation of ILC biology for the treatment of infectious and autoimmune disease.

This special issue of *Parasite Immunology* brings together recent insights from research on parasites that have advanced understanding of origins and functions of ILCs.

The discovery of ILCs has rewritten the immunology textbook. Evidence compiled over the last decade has described ILCs as new cell lineages that likely evolved as innate lymphoid precursors to the expansion of a functional adaptive response mediated by T‐cell populations. It is noteworthy that parasites were of fundamental importance to the initial identification of ILCs and subsequent discoveries of their functional roles in immunity. Thus, ILC discovery is not only another example of the field of immunoparasitology advancing our fundamental understanding of immunity, but a reminder that parasites wrote the original draft of the textbook of immunology during coevolution with humans. This is illustrated by the discovery of group 2 ILC in 2010, when independent groups published their studies describing a new class of innate lymphocyte with roles in initiation and maintenance of type 2 immune responses. Notably, all these studies used helminth infection models to expand this rare cell subset and to further explore cell function. Their findings demonstrated that these cells expand rapidly upon helminth infection, in response to IL‐25 and IL‐33, and are key to induction of archetypal anti‐helminth type 2 immune responses including goblet cell hyperplasia and mucus production. More recently, the same hookworm models led to further discoveries, including evidence of ILC2‐Th2 crosstalk and elucidation of novel functions of Tuft cells. The family of innate lymphoid cells now encompass ILC1, ILC2 and ILC3, each with distinct transcription factors and cytokine signatures (Figure 1).

**Figure 1:**
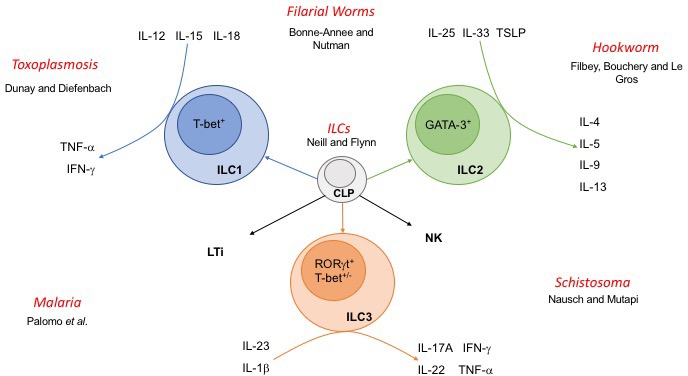
**Innate lymphoid cells and their interactions with parasites**. Innate lymphoid cells (ILCs) arise from a common lymphoid progenitor (CLP) and are delineated as ILC1, 2 or 3 based upon expression of lineage‐specific transcription factors and production of key cytokines. Natural killer (NK) and lymphoid tissue inducer (LTi) cells also arise from CLP and share some functional characteristics with ILCs. Parasites covered in this special issue are highlighted in red, with the names of the contributing authors.

In this special issue, we explore the roles of ILCs in defence against a number of parasitic infections (Figure 1). We begin with a reflection on the shared evolutionary history of ILCs and parasites. In their review, Neill and Flynn^1^ highlight recent advances in our understanding of the role of ILCs at barrier surfaces and argue that these functions are a consequence of evolution in the context of repeated pathogen exposure at these sites. As tissue‐resident cells, ILCs act as environmental sensors and first responders. Changes in ILC function in response to fluctuations in nutrient availability or disturbance of the microbiome may have evolved as a means of detecting and responding to the presence of parasites. This article highlights how parasites, in particular focusing on helminths in ILC2 biology, have profoundly influenced the mammalian immune system, the immunome, and perhaps have especially influenced ILC biology.

Our remaining reviews provide an overview of ILC research in the parasitology field. We cover protozoan and metazoan parasites, reviewing key studies in both mice and humans. Although ILC2s inevitably take a starring role, we highlight key interactions of both ILC1 and ILC3 with major parasites of humans.

In their review, Dunay and Diefenbach^2^ describe recent advances in our understanding of the roles of innate cells in *Toxoplasma gondii* infection. They describe a need for a reappraisal of the antiparasite roles attributed to NK cells in the light of recent findings with ILCs. Prior to the emergence of the ILC paradigm, widespread use of a NKp46^+^NK1.1^+^CD3e^−^ surface marker profile to identify NK cells may have led to the erroneous attribution of effector functions that are mediated by ILC3 or ILC1 to NK cells. Indeed, the authors’ recent work in mouse models suggests that ILC1s are the main producers of protective IFN‐γ and TNF‐α in early infection, with NK cells playing only a minor role.

Palomo et al^3^ explore emerging data describing a role for ILC2s in cerebral malaria. IL‐33 expands ILC2 and prevents experimental cerebral malaria in mice via a mechanism dependent on M2 macrophages and T regulatory cells. The authors highlight the disease‐resistant phenotype of PKCθ knockout mice, further suggesting a potential role for ILC1s in pathology. Finally, they identify a need for comprehensive elucidation of the role of ILCs in human cerebral malaria, given the evidence from controlled human malaria infection studies demonstrating changes in innate‐like lymphocytes.

Nausch and Mutapi^4^ take us from the protozoa to trematodes, and human helminth infections. ILC2 function in Schistosomiasis is described alongside a reflection on the implications of emerging paradigms in ILC biology on the design of helminth vaccines. The authors argue that induction of ILC2 responses should be considered in helminth vaccine design given their key role as a bridge between innate and adaptive immunity in helminth infection. In particular, ILC2 responses could be utilized as part of an infection and treatment strategy, whereby treatment with ILC2‐promoting agents such as IL‐2 or IL‐7 could be used to boost immune responses to vaccination.

Bonne‐Annee and Nutman^5^ bring together clinical studies and mouse models to provide an overview of ILC roles in filarial infection. While mouse model data have demonstrated the multifaceted role of ILC2 in control of *Litomosoides sigmodontis* infection, including both control of microfilaria and modulation of the humoral response, data are only now beginning to emerge from studies in humans. It is described how ILC frequencies are increased in filarial‐infected individuals and how such cells display an altered transcriptional profile. The authors highlight that unravelling the roles of ILCs in immunity to the various stages of the parasite lifecycle will be a major challenge.

Finally, Filbey, Bouchery and Le Gros^6^ take us back to where it all began, with a review on ILC2 functions in hookworm infection. The authors expand on the early discoveries regarding initiation of type 2 immune responses by ILC2 and provide an overview of recent advances in our understanding of ILC2 interplay with the adaptive immune system. Furthermore, they describe the key role of ILC2s in limitation and resolution of tissue damage following infection. Given the bystander damage caused by migration of large helminths through tissue, this role of ILCs may be another example of how the coevolution of these cells with parasites has shaped their functionality.

Our understanding of ILC biology has progressed at a remarkable pace over the last decade. Many of the key breakthroughs have come through investigation of ILC interactions with parasites, and parasitologists continue to drive the field forwards. However, key challenges remain, not least in reproducing key observations from animal parasite infection models in human disease. Indeed, while ILCs have been shown to have obligatory functions in experimental animal models, such as parasite infectivity, it remains to be seen whether they are functional or redundant in humans. This special issue highlights the significant contributions of immunoparasitology to the initial discovery and subsequent elucidation of functional roles of ILC, identifies future research directions and examines opportunities to harness advances in understanding of ILC biology for the betterment of human health, within parasitology and beyond.
